# Hypoxia Suppresses Spontaneous Mineralization and Osteogenic Differentiation of Mesenchymal Stem Cells via IGFBP3 Up-Regulation

**DOI:** 10.3390/ijms17091389

**Published:** 2016-08-24

**Authors:** Ji Hye Kim, Sei Mee Yoon, Sun U. Song, Sang Gyu Park, Won-Serk Kim, In Guk Park, Jinu Lee, Jong-Hyuk Sung

**Affiliations:** 1 College of Pharmacy, Yonsei Institute of Pharmaceutical Sciences, Yonsei University, Incheon 21983, Korea; wisdom-ks@hanmail.net (J.H.K.); sei_mee@naver.com (S.M.Y.); 2 Stemore Co., Ltd., Incheon 21983, Korea; 3 Department of Integrated OMICS for Biomedical Sciences, Yonsei University, Seoul 03722, Korea; 4 Inha University School of Medicine, Translational Research Center and Inha Research, Institute for Medical Sciences, Incheon 21983, Korea; sunuksong@inha.ac.kr; 5 College of Pharmacy, Ajou University, Suwon 16499, Korea; sgpark@ajou.ac.kr; 6 Department of Dermatology, Kangbuk Samsung Hospital, Sungkyunkwan University School of Medicine, Seoul 03722, Korea; susini@naver.com; 7 Department of Biochemistry, Stony Brook University, Stony Brook, NY 11790, USA; allpleache@naver.com

**Keywords:** adipose-derived stem cells, hypoxia, IGFBP3, reactive oxygen species, osteogenic differentiation

## Abstract

Hypoxia has diverse stimulatory effects on human adipose-derived stem cells (ASCs). In the present study, we investigated whether hypoxic culture conditions (2% O_2_) suppress spontaneous mineralization and osteogenic differentiation of ASCs. We also investigated signaling pathways and molecular mechanisms involved in this process. We found that hypoxia suppressed spontaneous mineralization and osteogenic differentiation of ASCs, and up-regulated mRNA and protein expression of Insulin-like growth factor binding proteins (IGFBPs) in ASCs. Although treatment with recombinant IGFBPs did not affect osteogenic differentiation of ASCs, siRNA-mediated inhibition of IGFBP3 attenuated hypoxia-suppressed osteogenic differentiation of ASCs. In contrast, overexpression of IGFBP3 via lentiviral vectors inhibited ASC osteogenic differentiation. These results indicate that hypoxia suppresses spontaneous mineralization and osteogenic differentiation of ASCs via intracellular IGFBP3 up-regulation. We determined that reactive oxygen species (ROS) generation followed by activation of the MAPK and PI3K/Akt pathways play pivotal roles in IGFBP3 expression under hypoxia. For example, ROS scavengers and inhibitors for MAPK and PI3K/Akt pathways attenuated the hypoxia-induced IGFBP3 expression. Inhibition of Elk1 and NF-κB through siRNA transfection also led to down-regulation of IGFBP3 mRNA expression. We next addressed the proliferative potential of ASCs with overexpressed IGFBP3, but IGFBP3 overexpression reduced the proliferation of ASCs. In addition, hypoxia reduced the osteogenic differentiation of bone marrow-derived clonal mesenchymal stem cells. Collectively, our results indicate that hypoxia suppresses the osteogenic differentiation of mesenchymal stem cells via IGFBP3 up-regulation.

## 1. Introduction

Hypoxia has diverse effects on human adipose-derived stem cells (ASCs) [1,2,3,4]. For example, hypoxia increased the expression of Oct4 and Rex1, and inhibited the aging/senescence of ASCs during culture [5]. Hypoxia also exhibited mitogenic effects, and it increased the proliferation and migration of ASCs [1,3,6]. Reactive oxygen species (ROS) are generated by NADPH oxidase (Nox) under hypoxic conditions in ASCs, and these signaling molecules mediate proliferation and migration [4]. Hypoxia-generated ROS increases the phosphorylated platelet-derived growth factor receptor-β PDGFR-β, and activated the Akt and ERK pathways [2,3,7,8]. Activation of these pathways induces NF-κB and ElK1 phosphorylation to up-regulate miR-210. Up-regulation ofmiR-210 down-regulates PTPN2 expression and increases the proliferation and migration of ASCs [9]. In addition, hypoxia significantly increases the secretion of diverse growth factors from ASCs, which collectively enhances the wound-healing and hair-regenerative potential of ASCs [6,10]. Therefore, it is plausible that hypoxia has beneficial effects on the stemness, self-renewal, proliferation, and regenerative potential of ASCs.

Hypoxia also affects ASC transdifferentiation [11,12,13,14]. For example, hypoxia generates ROS, and induces adipocyte differentiation of ASCs in adipocyte induction medium [11]. It has also been shown that hypoxia increases chondrogenic differentiation of ASCs. For example, hypoxic conditions increased hypoxia-inducible transcription factor 2α (HIF-2α) and enhanced chondrogenesis of ASCs from osteoarthritis patients [15]. The expression of chondrogenic markers such as COL2A1 and aggrecan was up-regulated when ASCs were exposed to hypoxia in chondrogenic medium [16]. However, hypoxia exhibited inhibitory effects on the osteogenic differentiation of ASCs [17,18]. With the presence of osteogenic medium under hypoxic conditions, osteocalcin expression, mineralization, and ALP activity were significantly decreased [16]. Expression of osteogenic markers revealed temporal changes of the osteogenic differentiation program in ASCs under different oxygen conditions [19]. Hypoxia (1% and 2% O_2_) also caused the osteogenic markers (ALP activity, mineralization, osteonectin, and osteopontin) to down-regulate in 2-D and 3-D culture, however; the inhibition of the HIF-1 activity only had a trivial impact on the expression of the osteogenic markers, indicating HIF-1-independent inhibition of osteogenic differentiation [12]. Hypoxia inhibited the metabolic switch (i.e., anaerobic metabolism was augmented) and mitochondrial function of bone marrow-derived mesenchymal stem cells (BM-MSCs), which also indicates suppression of osteogenic differentiation [17].

ASCs are popular seed cells for regenerative medicine, and ASC-based clinical trials are rapidly increasing. However, for their use in clinical trials, ASCs must first be expanded in vitro. During culture, ASCs may age owing to exposure to harmful conditions. For example, there was a report about spontaneous calcification of BM-MSCs from rats, which were under normoxia condition (20% O_2_) without osteogenic medium after continuous culture for 21 days [20]. Mineralized nodules were observed, which were positive for Alizarin Red, alkaline phosphatase (ALP), collagen, and osteocalcin. Spontaneous osteogenesis of BM-MSCs was also reported in cultured microcarriers through alteration of cytoskeletal tension [21]. Surprisingly, under hypoxia (2% O_2_) condition, spontaneous calcification was severely inhibited. In addition, the ALP and calcium content, BM-MSCs were sharply reduced [20]. These results suggest the importance of culturing ASCs and BM-MSCs under hypoxia, as this condition inhibits premature senescence of these stem cells. However, the underlying molecular mechanisms have not been identified yet. Therefore, we first examined whether hypoxic culture conditions (2% O_2_) suppress the spontaneous mineralization and osteogenic differentiation of ASCs. Next, we investigated signaling pathways and molecular mechanisms involved in this process.

## 2. Results and Discussion

### 2.1. Hypoxia Suppresses Spontaneous Mineralization and Osteogenic Differentiation of ASCs

We measured the effect of hypoxia (2% O_2_) on the mineralization and osteogenic differentiation of ASCs. ASCs were incubated in Minimum Essential Medium α (α-MEM) and osteogenic differentiation medium (ODM) for 7 and 14 days, and stained with Alizarin Red S (ARS).

**Figure 1:**
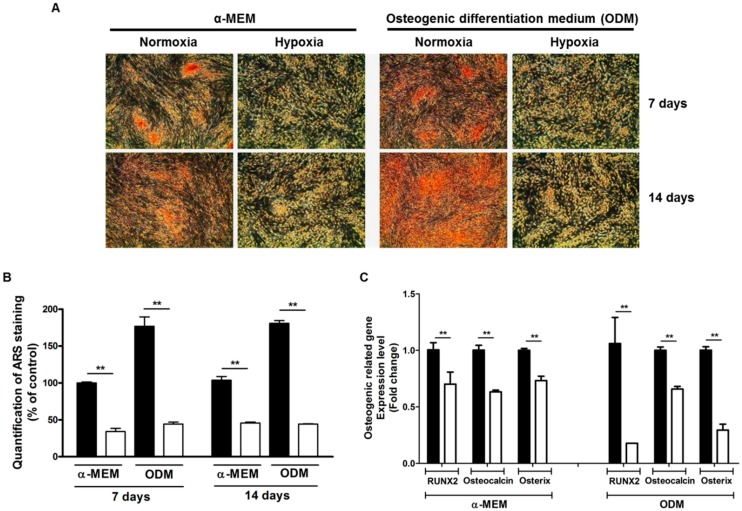
Hypoxia suppresses the spontaneous mineralization and osteogenic differentiation of ASCs. ASCs were cultured in α-MEM and osteogenic differentiation medium (ODM) for 7 and 14 days, and then stained with Alizarin Red S (ARS). (**A**,**B**) Hypoxia (2% O_2_) significantly reduced the ARSstaining of ASCs (red) in α-MEM and ODM (40×), and ARS staining was measured; (**C**) osteogenic differentiation markers such as RUNX2, osteocalcin, and osterix were significantly reduced under hypoxia. Normoxia: black bars, Hypoxia: white bars. ** *p* *<* 0.01.

Hypoxia significantly reduced the spontaneous mineralization and osteogenic differentiation of ASCs (Figure 1A). ARS staining of ASCs was quantified in α-MEM and ODM (Figure 1B). In addition, osteogenic induction markers such as RUNX2, osteocalcin, and osterix were significantly reduced under hypoxia (Figure 1C). Collectively, these results indicate that hypoxia suppress the spontaneous mineralization and osteogenic differentiation of ASCs.

### 2.2. Hypoxia Induces IGFBP Expression

**Figure 2:**
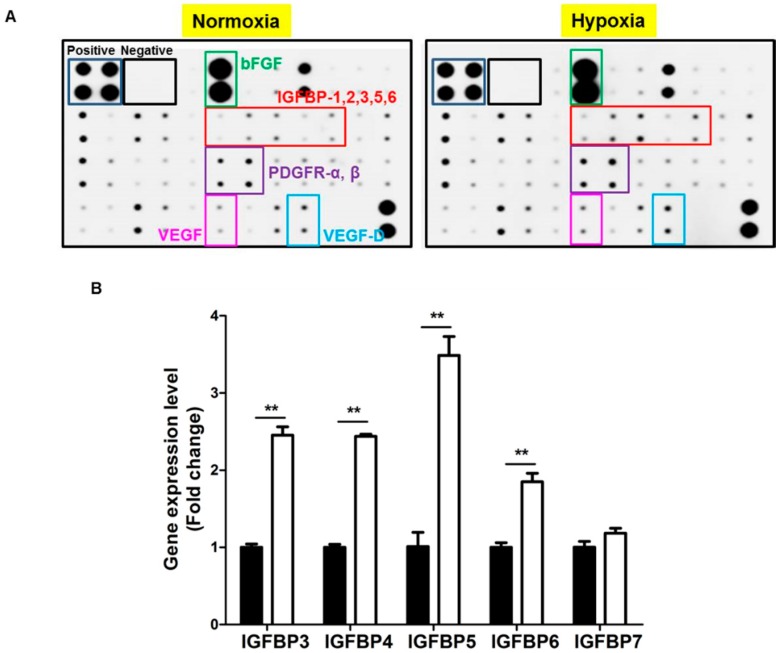
Hypoxia induces IGFBP expression. (**A**) The altered expression of growth factors was examined using an antibody array. Hypoxia (2%, 24 h) upregulated the protein expression of IGFBP families in ASCs; (**B**) the mRNA level of IGFBP families under hypoxia was measured. Hypoxia significantly upregulated IGFBP3–6. Normoxia: black bars, hypoxia: white bars. ** *p* < 0.01.

We have previously reported that hypoxia up-regulates diverse growth factors expression [1,2,22,23]. We therefore examined the altered expression of growth factors by antibody array, and found that hypoxia up-regulated the protein expression of IGFBP families in ASCs (Figure 2A). We further examined the mRNA expression of IGF, IGF receptor and IGFBP families in ASCs. As summarized in Table 1, IGF-1, IGF-2, and their receptors are poorly expressed in ASCs. IGFBP1 and IGFBP2 are not expressed; however, IGFBP3–7 is highly expressed in ASCs. Therefore, we measured the mRNA expression of IGFBP3–7 under hypoxia, which led to significant up-regulation of IGFBP3–6 (Figure 2B).

### 2.3. Recombinant IGFBP3–6 Does Not Suppress the Osteogenic Differentiation of ASCs

Since hypoxia up-regulates IGFBP expression, we treated ASCs with recombinant IGFBP3–6 to measure the effects on osteogenic differentiation. However, IGFBP3–5 (at 10–1000 ng/mL) did not alter the osteogenic differentiation of ASCs (Figure S1). In contrast, IGFBP6 induced osteogenic differentiation of ASCs at high concentration (Figure S1B).

### 2.4. Role of Intracellular IGFBP3 in Osteogenic Differentiation

**Figure 3:**
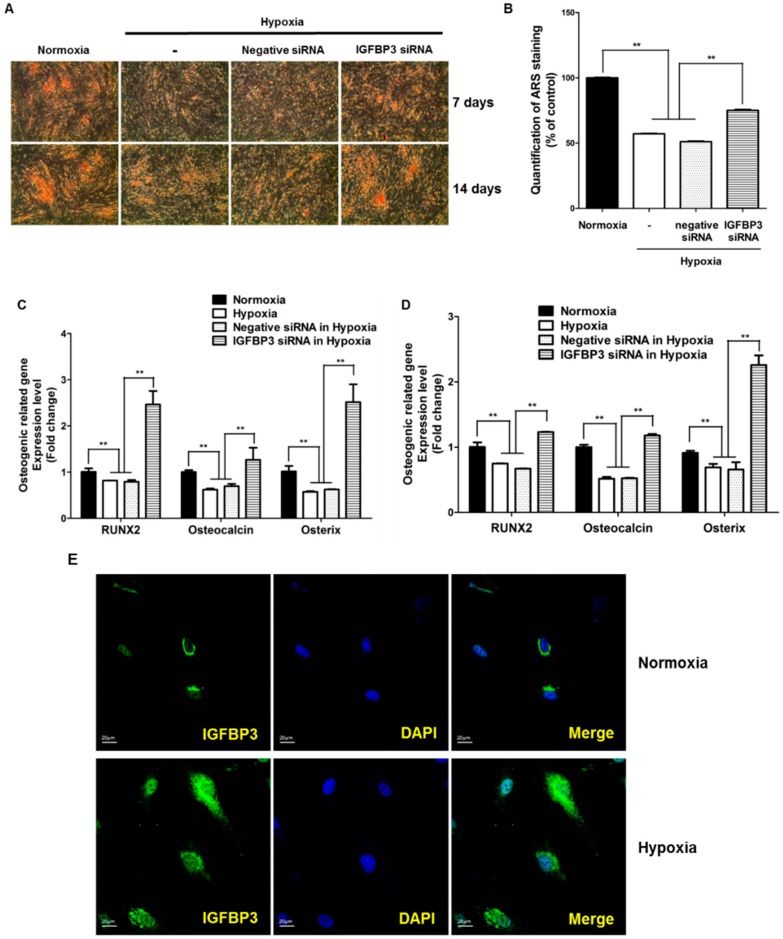
Intracellular IGFBP3 inhibits osteogenic differentiation. (**A**) siRNA knockdown of IGFBP3 induced ARS staining in ASCs at 7 and 14 days (40×); (**B**) quantification of ARS staining was measured at 7 days; (**C**,**D**) siRNA knockdown of IGFBP3 significantly attenuated hypoxia-reduced osteogenic induction markers such as RUNX2, osteocalcin, and osterix at 7 and 14 days; (**E**) hypoxia increased the transfer of IGFBP3 into the nuclear region of ASCs, and IGFBP3 signal (green) is increased in the nuclear region (DAPI, blue) of ASCs. ** *p* < 0.01.

Since exogenous recombinant IGFBPs did not affect the osteogenic differentiation of ASCs, we further examined whether endogenous intracellular IGFBPs affected osteogenic differentiation. We thus examined whether siRNA-mediated inhibition of IGFBP proteins could attenuate hypoxia-induced suppression of osteogenic differentiation. We prepared siRNAs of IGFBP3–5, and transfected them into ASCs. siRNA transfection reduced the mRNA expression of IGFBP3–5 in ASCs (Figure S2A,B). Compared with other IGFBPs, IGFBP3 plays a pivotal role in osteogenic differentiation of ASCs (Figure S2C). For example, knockdown of IGFBP3 led to positive ARS staining in ASCs at 7 and 14 days (Figure 3A,B). In addition, inhibition of IGFBP3 significantly up-regulated the hypoxia-reduced osteogenic markers such as RUNX2, osteocalcin, and osterix at 7 and 14 days (Figure 3C,D). We also examined whether hypoxia increases the translocalization of IGFBP3 into the nuclear region of ASCs. Compared to controls, IGFBP3 expression (green) was increased in the nuclear region (DAPI, blue) of ASCs under hypoxia (Figure 3E).

**Figure 4:**
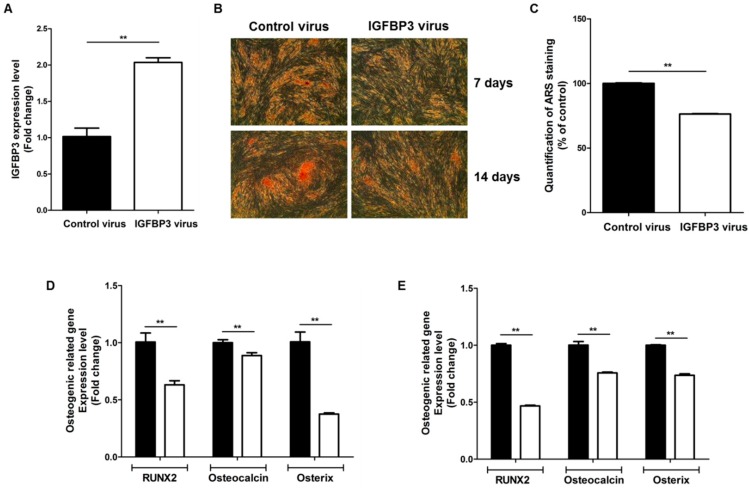
IGFBP3 overexpression reduces the osteogenic differentiation of ASCs. (**A**) Overexpression of IGFBP3 using lentiviral vectors; (**B**) IGFBP3 overexpression inhibited the osteogenic differentiation of ASCs at 7 and 14 days (40×); (**C**) quantification of ARS staining was measured at 7 days; (**D**,**E**) in addition, IGFBP3 overexpression significantly inhibited the expression of osteogenic induction markers such as RUNX2, osteocalcin, and osterix at 7 days (**D**) and 14 days (**E**). Control: black bars, IGFBP3 overexpression: white bars. ** *p* < 0.01.

In addition, we examined the gain-of-function by IGFBP3 overexpression. Transfection of IGFBP3 with lentiviral vectors significantly up-regulated IGFBP3 expression (Figure 4A), and IGFBP3 overexpression inhibited the osteogenic differentiation of ASCs at 7 and 14 days (Figure 4B,C). In addition, IGFBP3 overexpression significantly inhibited the expression of osteogenic induction markers such as RUNX2, osteocalcin, and osterix at 7 days (Figure 4D) and 14 days (Figure 4E).

### 2.5. ROS Up-Regulates IGFBP3 Expression

**Figure 5:**
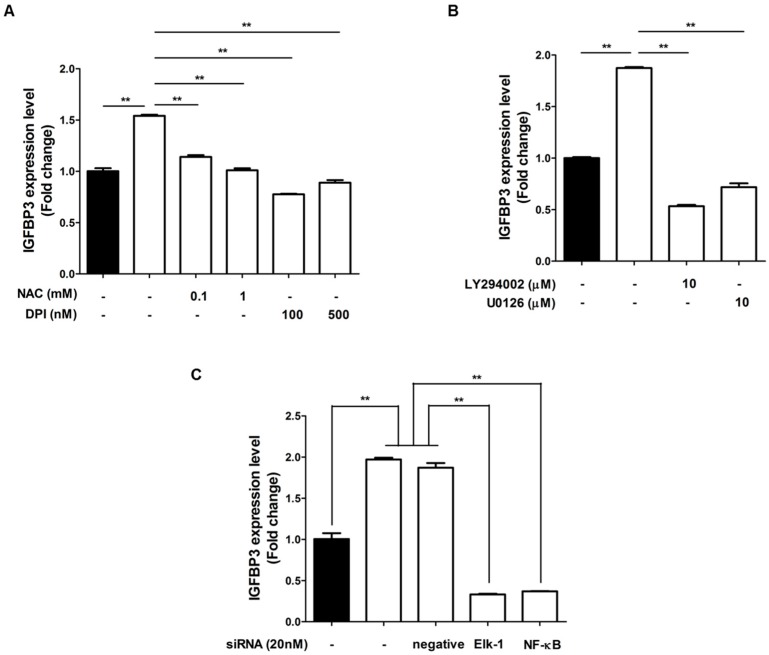
ROS up-regulates IGFBP3 expression. (**A**) mRNA expression of IGFBP3 was measured after NAC or DPI treatment. These treatments attenuated hypoxia-induced IGFBP3 upregulation; (**B**) chemical inhibition of Akt and ERK by LY294002 and U0126, respectively, significantly reduced the hypoxia-induced IGFBP3 mRNA level; (**C**) siRNA transfection of NF-κB and Elk1 significantly attenuated the IGFBP3 levels under hypoxia. Normoxia: black bars, hypoxia: white bars. ** *p* < 0.01.

We previously demonstrated that certain growth factor expression in ASCs was mediated by ROS generation [8]. Therefore, we investigated whether IGFBP3 expression is regulated by ROS signaling. First, we measured the mRNA expression of IGFBP3 after *N*-acetyl-cysteine (NAC, ROS scavenger) or diphenyleneiodonium chloride (DPI, Noxinhibitor) treatment, which attenuated hypoxia-induced IGFBP3 up-regulation (Figure 5A). These results indicate that ROS generation under hypoxia mediates IGFBP3 expressionin ASCs. Since ROS generation primarily induced Akt and ERK phosphorylation, followed by NF-κB and Elk1 phosphorylation, we detected the alteration of IGFBP3 expression using inhibitors of these signaling pathways [2,9]. Chemical inhibition of Akt and ERK by LY294002 and U0126, respectively, significantly reduced hypoxia-induced IGFBP3 mRNA levels (Figure 5B). In addition, siRNA transfection of NF-κB and Elk1 significantly attenuated IGFBP3 levels under hypoxia (Figure 5C). These results collectively indicate that ROS generation, as well as the PI3K/Akt and MAPK pathways are involved in IGFBP3 expression under hypoxia.

### 2.6. Hypoxia Suppresses the Osteogenic Differentiation of Clonal BM-MSCs via IGFBP3

**Figure 6:**
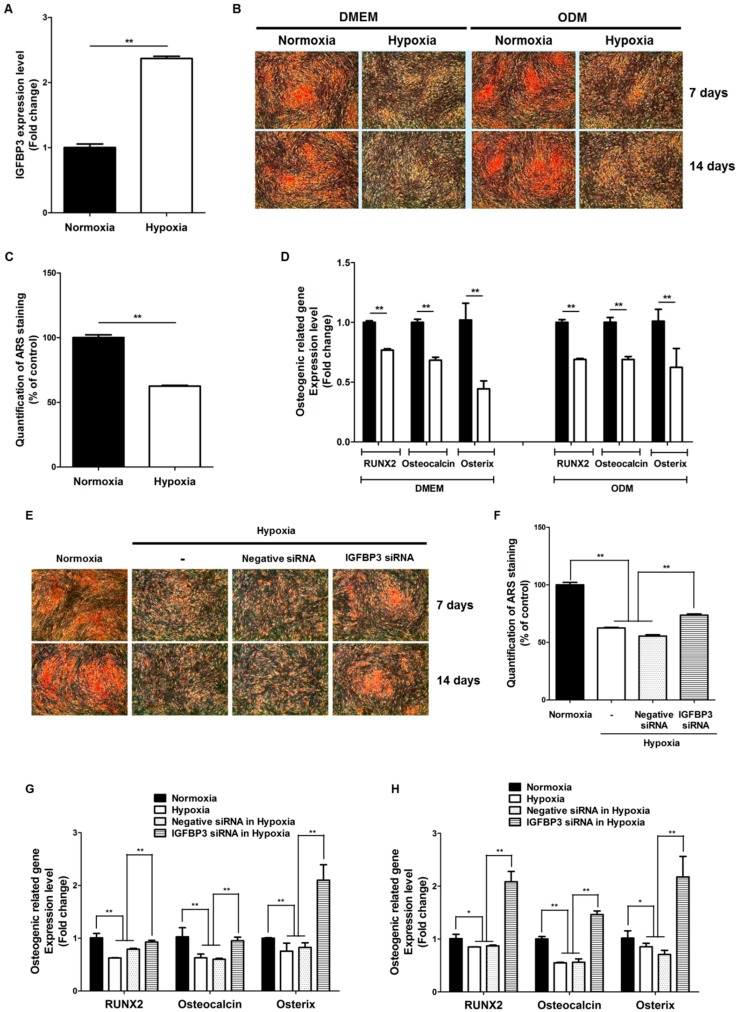
Hypoxia suppresses the osteogenic differentiation of clonal BM-MSCs. (**A**) Hypoxia (2%) induced the mRNA expression of IGFBP3 in clonal BM-MSCs; (**B**) hypoxia suppressed the osteogenic differentiation of clonal BM-MSCs at 7 and 14 days (40×); (**C**) quantification of ARS staining was measured at 7 days; (**D**) osteogenic induction markers such as RUNX2, osteocalcin, and osterixwere significantly reduced by hypoxia in DMEM and ODM; (**E**) siRNA-mediated inhibition of IGFBP3 significantly attenuated the hypoxia-suppressed osteogenic differentiation of clonal BM-MSCs at 7 and 14 days (40×); (**F**) quantification of ARS staining was measured at 7 days; (**G**,**H**) osteogenic induction markers such as RUNX2, osteocalcin, and osterix were significantly reduced by hypoxia at 7 days (**G**) and 14 days (**H**). Normoxia: black bars, hypoxia: white bars. * *p* < 0.5, ** *p* < 0.01.

Since osteogenic differentiation is also important for BM-MSCs, we examined the effect of hypoxia on their osteogenic differentiation, as well as the involvement of IGFBP3 during osteogenesis. We used clonal BM-MSCs derived using our previously reported novel method of isolation [24,25] to examine the effect of hypoxia on osteogenic differentiation. As expected, hypoxia (2%) induced the mRNA expression of IGFBP3 in clonal BM-MSCs (Figure 6A). Hypoxia suppressed the osteogenic differentiation of clonal BM-MSCs at 7 and 14 days (Figure 6B). Osteogenic induction markers such as RUNX2, osteocalcin, and osterix were significantly reduced by hypoxia in DMEM and ODM (Figure 6D). In addition, siRNA-mediated inhibition of IGFBP3 significantly attenuated the hypoxia-suppressed osteogenic differentiation of clonal BM-MSCs at 7 and 14 days (Figure 6E,F). Osteogenic induction markers such as RUNX2, osteocalcin, and osterix were significantly reduced by hypoxia at 7 days (Figure 6G) and 14 days (Figure 6H).

## 3. Discussion

In the present study, we examined whether hypoxic culture conditions (2% O_2_) suppress spontaneous mineralization and osteogenic differentiation of ASCs. We further investigated the signaling pathways and molecular mechanisms involved in the suppression of spontaneous mineralization and osteogenic differentiation of ASCs under hypoxia. We found that hypoxia suppressed the spontaneous mineralization and osteogenic differentiation of ASCs and up-regulated the mRNA and protein expression of IGFBPs in ASCs. Although treatment with recombinant IGFBPs did not affect the osteogenic differentiation of ASCs, inhibition of IGFBP3 via siRNA attenuated hypoxia-suppressed osteogenic differentiation of ASCs. In contrast, overexpression of IGFBP3 via lentiviral vector inhibited their osteogenic differentiation. These results indicate that hypoxia suppresses the spontaneous mineralization and osteogenic differentiation of ASCs via IGFBP3 up-regulation. We also investigated the effect of related signaling pathways, demonstrating that ROS generation under hypoxia plays a pivotal role in activation of the MAPK and PI3K/Akt pathway. In addition, siRNA inhibition of Elk1 and NF-κB down-regulated mRNA expression levels of IGFBP3 under hypoxia, and regulate its transcription. In short, intracellular IGFBP3 expression increased and IGFBP3 translocated to the nuclear region of ASCs, and intracellular IGFBP3 inhibited the spontaneous mineralization and osteogenic differentiation of ASCs under hypoxia. Furthermore, we found that hypoxia suppressed the osteogenic differentiation of clonal BM-MSCs via IGFBP3 overexpression.

Wang et al. observed that momentary exposure of BM-MSCs after osteogenic induction for 7 days under hypoxia (2% O_2_) condition led to decreased ALP activity, osteocalcin and Runx2/Cbfa1 expression [26]. Hypoxia condition causes an early and transient increment of the phosphorylated ERK1/2 levels. This outcome suggests that hypoxia could suppress osteogenic differentiation of BM-MSCs possibly through the MEK-ERK1/2 pathway [26]. Ismail et al. reported that the hypoxia-induced proliferation of human pulmonary artery smooth muscle cells (HPASMCs) is mediated by NADPH oxidase 4 (NOX4) through the autocrine production of transforming growth factor-β1 and IGFBP3 [27]. HPASMCs produce TGF-β1, which acts as an autocrine factor to induce IGFBP3 through the PI3K/Akt pathway under hypoxia [27]. In the present study, we found that ROS mediated IGFBP3 expression in ASCs under hypoxia (2% O_2_). ROS generation activated the MAPK and PI3K/Akt pathways, which are involved in the upregulation of IGFBP3 in ASCs. We also identified the transcription factors for IGFBP3 expression under hypoxia. Down-regulation of Elk1 and NF-κB by siRNA technic attenuated IGFBP3 upregulation, which indicates that Elk1 and NF-κB are possible transcription factors for IGFBP3. Collectively, these results indicate that MAPK and PI3K/Akt pathways play a pivotal role in the IGFBP3 upregulation and the suppression of osteogenic differentiation of ASCs under hypoxia.

It has been reported that IGF1 and IGF2 induce osteogenic differentiation [28,29,30]. For example, IGF2 enhances bone formation and BMP9-induced osteogenic differentiation, and BMP9 crosstalks with IGF2 through the PI3K/Akt signaling pathway in the time of osteogenic differentiation of MSCs [28]. Because IGFBPs bind IGFs to inhibit their actions, we first hypothesized that IGFBPs suppress osteogenic differentiation of ASCs by antagonizing IGFs. Therefore, we treated ASCs with recombinant IGFBP3–6 with or without osteogenic differentiation medium in a preliminary study (Figure S1). However, recombinant IGFBP did not inhibit the spontaneous calcification and osteogenic differentiation of ASCs (Figure S1). Instead, inhibition of intracellular IGFBP3 by siRNA transfection and its overexpression modulate the osteogenic differentiation of ASCs. These results indicate that secreted IGFBPs from ASCs do not have an effect on osteogenic differentiation, but intracellular IGFBPs can have an impact under hypoxia.

In addition to its actions outside the cell, cellular uptake and nuclear import of IGFBP3 has been known about for almost two decades [31,32]. In the nucleus, IGFBP3 is involved in transcription regulation. IGFBP3 can bind to the nuclear receptor and some of its dimerization partners, such as peroxisome proliferator-activated receptor-γ (PPAR-γ), retinoic acid receptor (RAR), and vitamin D receptor (VDR). These interactions adjust the functions of these receptors; for example, inhibition of PPAR-γ-dependent transcription in adipocytes [32,33]. IGFBP3 also modulated osteoblast differentiation via interaction with VDR [34]. IGFBP3 prevented RAR heterodimerization, and modulated retinoic acid-sensitivity in human cancer cells [35,36]. In short, IGFBP3 can influence cellular function by interacting with intranuclear pathways. In the present study, we investigated the interaction of IGFBP3 and VDR under hypoxia, showing that IGFBP3 does not colocalize with VDR in the nuclear region (Figure S3). IGFBP3 also did not interact with VDR in an immunoprecipitation assay (Figure S3). It is thus reasonable to assume that suppression of spontaneous calcification and osteogenic differentiation of ASCs under hypoxia is not mediated by VDR.

IGFBP3, a hypoxia-inducible gene, regulates various cellular processes, such as apoptosis, cell proliferation, epithelial-mesenchymal transition and senescence. In addition, it has been related to pathogenesis of cancers [37,38]. IGFBP3 induced cancer cell proliferation and thus can be used in cancer diagnosis [39,40]. However, there are several controversial reports on the apoptotic effect of IGFBP3 in various cell types [38,41]. Nuclear IGFBP3 can be observed by immunohistochemistry in cancer and other tissues. In many cell culture studies, it is necessary in the nucleus because of its pro-apoptotic effect, which may involve interaction with the nuclear receptor Nur77 [42,43]. In the present study, we overexpressed IGFBP3 in ASCs and investigated its mitogenic effects in ASCs. IGFBP3 overexpression reduced the proliferation of ASCs in the present study (Figure S4). In addition, we examined the regenerative potential of ASC by IGFBP3 overexpression, but IGFBP transfection did not enhance the hair-regenerative potential of ASCs (Figure S4). Therefore, use of IGFBP3-overexpressed ASCs is not recommended as a potential method for enhancing the proliferation and regenerative ability of ASCs.

## 4. Materials and Methods

### 4.1. Materials

Chemicals such as 0.1 and 1 mM NAC (Sigma-Aldrich, St. Louis, MO, USA), 100 and 500 nM DPI, 10 μM LY294002 (PI3K inhibitor, Calbiochem, San Diego, CA, USA), and 10 μM U0126 (ERK inhibitor, Calbiochem) were used in the inhibition study.

Antibodies recognizing IGFBP3 (1:200) were purchased from Santa Cruz Biotechnology (Santa Cruz, CA, USA). A fluorescein isothiocyanate (FITC, 1:500)-conjugated secondary antibody was purchased from Invitrogen (Carlsbad, CA, USA).

### 4.2. Cell Culture and Osteogenic Differentiation

Human ASCs were isolated via liposuction of subcutaneous fat, as described previously [4,7], after informed consent was obtained (Boondang CHA hospital, BD2011-152D). ASCs were cultured in α-MEM (Hyclone, Thermo Scientific, Logan, UT, USA) with 10% fetal bovine serum (FBS, GIBCO, Invitrogen) and 1% penicillin and streptomycin. Human clonal bone marrow-derived mesenchymal stem cells (cBM-MSCs) were obtained from SCM life science (Incheon, Korea), and maintained in Dulbecco’s modified Eagle’s medium (DMEM; Hyclone) with 10% FBS, 1% penicillin, and streptomycin. Cells were maintained at 37 °C under humidified 5% CO_2_ (normoxia), 2% O_2_, and balanced N_2_ (hypoxia) conditions. Characterization of ASCs was performed by cell surface marker staining for CD34, CD73, CD90, and CD105.

For osteogenic differentiation, cells were cultured in ODM containing 10% FBS, 50 μg/mL l-ascorbic acid, and 10 mM β-glycerophosphate. ODM was changed every 3 days.

### 4.3. Spontaneous Mineralization

Cells were seeded on a 12-well plate at a density of 3 × 10^4^ cells per well. After 7 or 14 days of culture, cells were stained by Alizarin Red S (ARS) or harvested for RNA expression.

### 4.4. Alizarin Red S Staining

For ARS staining, cells were rinsed with PBS and fixed with 4% paraformaldehyde at room temperature. After 10 min, cells were rinsed twice with PBS and stained with 2% ARS for 10 min. The cells were then washed twice with distilled water for 3 min. Mineralization was quantified using 10 mM sodium phosphate (pH 7.0) and 10% cetylpyridinium chloride. After 15 min, the absorbance of the extracted solution was measured at 560 nm.

### 4.5. Membrane-Based Human Growth Factor Assay

ASCs were incubated under normoxia and hypoxia for 16 h, and cell lysate was used to detect the growth factor expression. Human Growth Factor Antibody Array kit was purchased from RayBiotech (Norcross, GA, USA), and antibody array was performed according to the manufacturer’s manual [10].

### 4.6. siRNA Transfection

IGFBP3 siRNA (15 nM) (Integrated DNA Technologies, Coralville, IA, USA) and Elk1 and NF-κBsiRNAs (20 nM each) were mixed with RNAi max before the cell trypsinization step. Cells were then seeded on 12-well plates or 60-mm dishes for 30 min. The siRNA mixture was then allowed to transfect cells for 48 h. Silencing was evaluated by RT-PCR.

### 4.7. IGFBP3 Overexpression

Plasmids containing the human IGFBP3 coding sequence (clone ID hMU011298) were provided by the Korea Human Gene Bank, Medical Genomics ResearchCenter, KRIBB, Korea. The IGFBP3 fragment was excised with *Eco*RI/*Bam*HI from the plasmid and inserted into pLVX-EF1α-IRES-Puro (Clontech, Mountain View, CA, USA) at *Eco*RI/*Bam*HI recognition sites, named pLVX-EIP-hIGFBP3. The empty vector, pLVX-EF1α-IRES-Puro, was used as a control. To produce lentiviruses, pLVX-EF1α-IRES-Puro or pLVX-EIP-hIGFBP3 combined with psPAX2 and pMD2.G, were mixed at a ratio of 4:3:1. A total of 3 μg DNA was transfected into HEK293T cells (ATCC, Manassas, VA, USA) grown to more than 80% confluence on 6-well plates with iN-fect^TM^ (iNtron Biotechnology, Seongnam, Korea), according to the manufacturer’s instructions. After 48 h of incubation, the conditioned media containing lentivirus were cleared with brief centrifugation and stored at −80 °C before use. To determine the lentivirus titer, p24 concentration was measured using the Lenti-X^TM^ p24 Rapid Titer Kit. Infectious titers of the control and IGFBP3 lentiviruses were approximated at 1.0 × 10^7^ and 1.1 × 10^7^ TU/mL, respectively using the manufacturer’s conversion guides.

For lentivirus transduction, cells were seeded on 12-well plates at a density of 1 × 10^4^ cells per well. After 24 h, medium was replaced with lentivirus and 4 μg/mL polybrene. The following day, the medium was replaced with complete media. Cells were then selected by 1 μg/mL of puromycin for 3 days.

### 4.8. Immunostaining of IGFBP3

For staining of IGFBP3, ASCs were seeded on a circular cover glass. The following day, cells were incubated in normoxia or hypoxia (2% O_2_) for 24 h. Then, cells were fixed using 4% paraformaldehyde for 15 min and permeabilized using 0.5% PBS-T for 5 min. After washing with 0.1% PBS-T, cells were blocked using blocking solution (10% FBS and 0.5% gelatin in PBS) for 1 h at room temperature. Primary antibody for IGFBP3 was diluted at 1:200 and the secondary antibody (FITC) at 1:500. For nuclear area staining, cells were treated and stained with DAPI. Fluorescence signals were detected using a confocal microscope (Carl Zeiss, Oberkochen, Germany).

### 4.9. Immunoprecipitation Assay

Cells were lysed with RIPA buffer (20 mM Tris-HCl, pH 7.6, 150 mM NaCl, 1 mM EDTA, 1% NP-40, 1% Sodium deoxycholate, 0.1% SDS, 10 mM β-glycerophosphate, 1 mM Sodium orthovanadate, 10 mM NaF, 1 mM PMSF, 1× protease inhibitor cocktail) and incubated on ice for 30 min. Whole cell lysates were prepared by centrifugation at 25,000× *g* for 10 min at 4 °C. The protein extracts (1 mg) were incubated with normal IgG (0.5 mg) or anti-IGFBP3 antibody (0.5 mg) for 14 h at 4 °C and then with protein A agarose for 2 h at 4 °C. The beads were washed three times with RIPA buffer, and resuspended in 1× SDS sample buffer. The samples were boiled and loaded into 10% SDS-PAGE for Western blot.

### 4.10. RNA Isolation and Quantitative Real-Time PCR

Total RNA was extracted using Trizol or QIAGEN RNA prep kit and isolated mRNA was reverse-transcribed using the cDNA synthesis kit (Promega, Madison, WI, USA). cDNA was synthesized from 500 ng of total RNA by 200 U reverse transcriptase and 50 ng/μL oligo (dT). Thermal cycling was performed over 35 cycles; reactions started at 95 °C for 5 min, then 95 °C for 30 s, 58 °C for 20 s, 72 °C for 40 s, and terminated at 72 °C for 5 min. Quantitative real-time PCR reactions were performed on Step one plus real-time PCR system (AB Applied Biosystems, Invitrogen) using SYBR green PCR master mix (TaKaRa, Otsu, Japan). The level of GAPDH or 18S rRNAwas used for sample standardization. Analysis of fold change was calculated by Δ*C*_t_ value.

### 4.11. Cell Proliferation

Stable cell lines overexpressing IGFBP3, generated through puromycin selection for 3 days, were seeded on 12-well plates at a density of 0.8 × 10^4^ cells per well. After 24 h, the medium was replaced and incubated in at 37 °C in a humidified 5% CO_2_ incubator for 48 h. ASC proliferation was measured by IncuCyte Zoom (ESSEN BioScience, Ann Arbor, MI, USA).

### 4.12. Animal Study

For hair regeneration experiment, 7 weeks old C_3_H/HeN mice were maintained according to Institutional Animal Care and Use Committee of Yonsei University (IACUC-A-201507-371-02, 2015/07/15). 1 × 10^4^ of cells of control vector transduced ASCs or IGFBP3 overexpressed ASCs were injected into shaved dorsal skin. After 14 days, hair regeneration was measured by hair weight of back skin.

### 4.13. Statistical Analysis

All data are representative of triplicate independent experiments. The statistical significance of the differences among groups was tested using the ANOVA or Student’s *t*-test. Values of *p* < 0.05 or *p* < 0.01 were considered significant.

## 5. Conclusions

We examined whether hypoxic culture conditions (2% O_2_) suppress spontaneous mineralization and osteogenic differentiation of ASCs, and investigated the signaling pathways and underlying molecular mechanisms. Our results indicate that hypoxia suppresses the osteogenic differentiation of ASCs via IGFBP3 upregulation. Hypoxia upregulates IGFBP3 expression through ROS generation, followed by activation of MAPK and PI3K/Akt pathways.

## Figures and Tables

**Table 1:** List of *IGFs*, *IGF receptors* and *IGFBPs* expression levels.

	Gene	*C*_t_ Value
1	*IGF1*	not detected
2	*IGF2*	31.55 (±0.35)
3	*IGF1R*	30.14 (±0.19)
4	*IGF2R*	not detected
5	*IGFBP1*	not detected
6	*IGFBP2*	not detected
7	*IGFBP3*	21.92 (±0.12)
8	*IGFBP4*	25.38 (±0.04)
9	*IGFBP5*	25.89 (±0.04)
10	*IGFBP6*	21.52 (±0.09)
11	*IGFBP7*	19.91 (±0.04)
12	*GAPDH*	19.61 (±0.08)
13	*18S*	24.74 (±0.08)

## Supplementary Materials

Supplementary materials can be found at www.mdpi.com/1422-0067/17/9/1389/s1.
