# Supplementary material for: Hypoxia Suppresses Spontaneous Mineralization and Osteogenic Differentiation of Mesenchymal Stem Cells via IGFBP3 Up-Regulation
Source: Int J Mol Sci. 2016 Aug 24;17(9):1389. doi: 10.3390/ijms17091389 (PMC5037669; doi:10.3390/ijms17091389)
Supplement: Supplementary file 1 [file ijms-17-01389-s001.pdf]

# Supplementary Materials: Hypoxia Suppresses Spontaneous Mineralization and Osteogenic Differentiation of Mesenchymal Stem Cells via IGFBP3 Up-Regulation

**Ji Hye Kim, Sei Mee Yoon, Sun U. Song, Sang Gyu Park, Won-Serk Kim, In Guk Park, Jinu Lee and Jong-Hyuk Sung**

## 1. Osteogenic Differentiation of ASCs Was Not Different by Exogenous IGFBP3–6 Recombinant Protein

For detection of recombinant IGFBP3–6 protein effect, ASCs were treated 10, 100, 500 and 1000 ng/mL of human IGFBP3–6 recombinant protein. After 7 days, ASCs were stained by Alizarin Red S. Osteogenic differentiation of ASCs was not altered by exogenous IGFBPs protein.

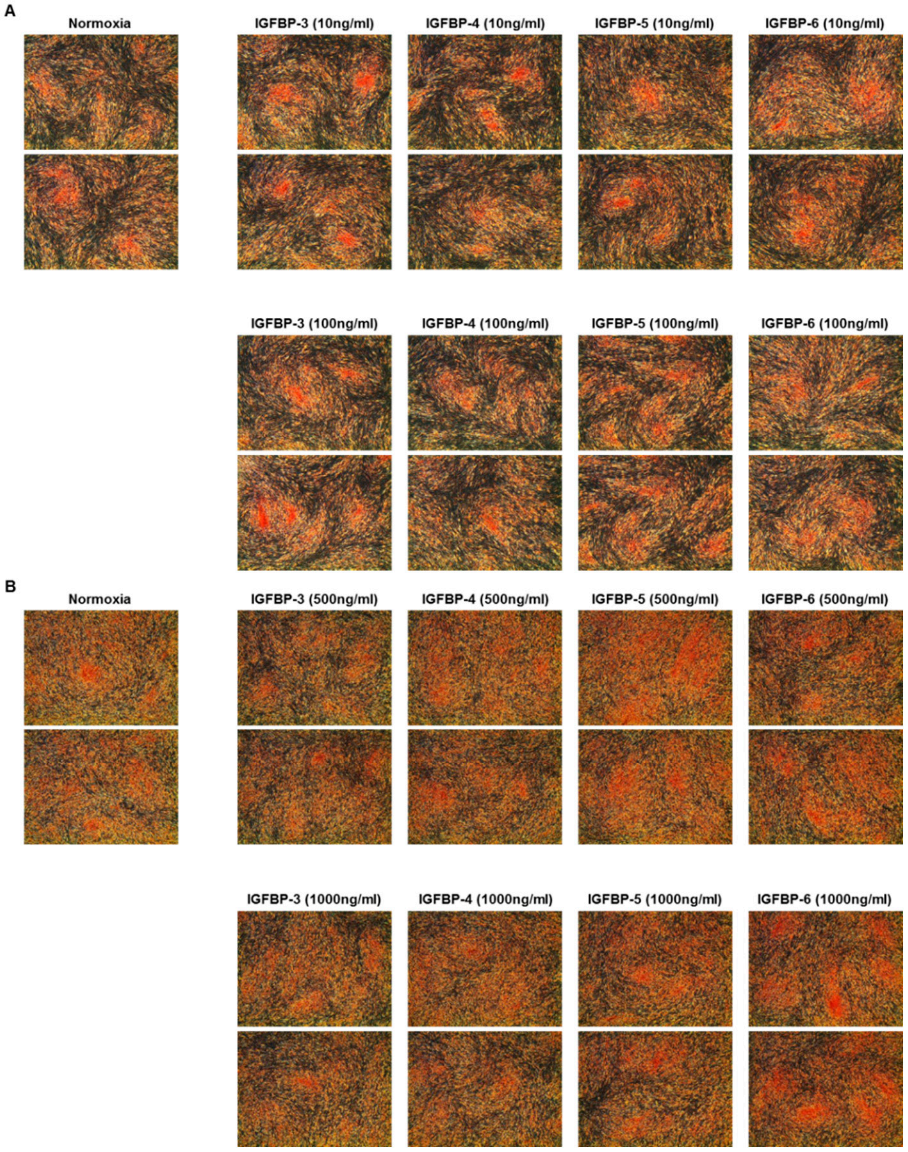


**Figure S1.** Osteogenic differentiation effect of exogenous IGFBP3–6. (**A**) ASCs were treated 10 and 100 ng/mL of IGFBP3–5 recombinant protein for 7 days; (**B**) ASCs were treated 500 and 1000 ng/mL of IGFBP3–5 recombinant protein. After 7 days, cells were detected by ARS staining (40×).

## 2. IGFBP3–5 Knockdown by siRNA Transfection

For IGFBP3–5 knockdown, ASCs were transfected siRNA with RNAi Max. We detected efficiency of siRNA using PCR (A) and real-time PCR (B). At result, gene expression of IGFBP3–5 was decreased by siRNA.

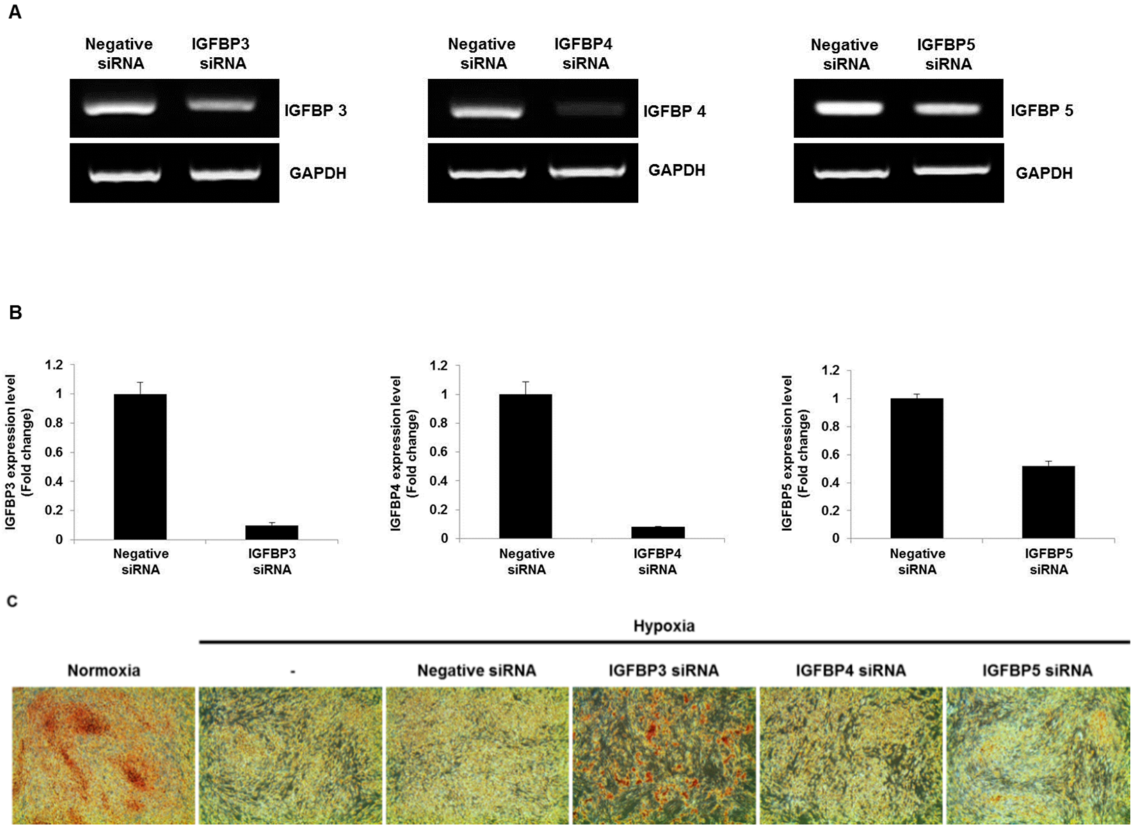


**Figure S2.** Knockdown of *IGFBP3–5* gene expression. 15 nM of IGFBP3 siRNA and 20 nM of IGFBP4–5 siRNA were treated in ASCs. For siRNA control, ASCs were treated negative siRNA. After 48 h, ASCs were harvested for RNA prep. *IGFBP3–5* expression level was detected by agarose gel (**A**) and quantitative real-time PCR (**B**); (**C**) ASCs were transfected with siRNA for 48 h and stained by ARS after 14 days (40×).

## 3. Interaction of IGFBP3 and Vitamin D Receptor

In previous studies, mechanism of osteogenesis related with nuclear receptors such as vitamin D receptor (VDR). To demonstrate interaction of IGFBP3 and VDR, we performed immunofluorescence and detected colocalization of IGFBP3 and VDR in the nucleus. IGFBP3 protein level was increased under hypoxia in the nucleus (Figure S3A,B, red signal). However, IGFBP3 and VDR signal only slightly merge under hypoxia in the nucleus (Figure S3B, red vs. green). In addition, IGFBP3 and VDR do not bind in immunoprecipitation (Figure S3C).

A

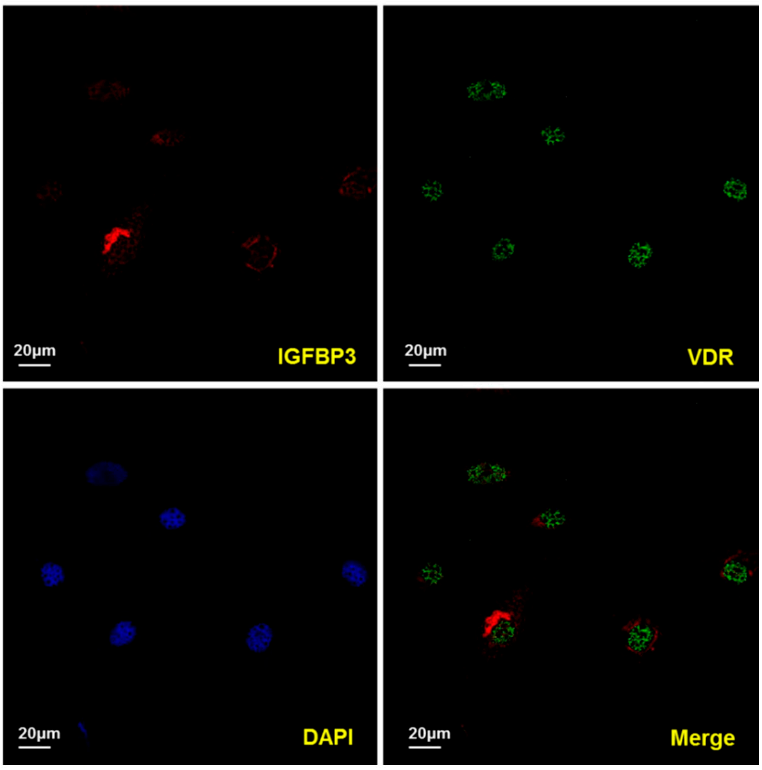


B

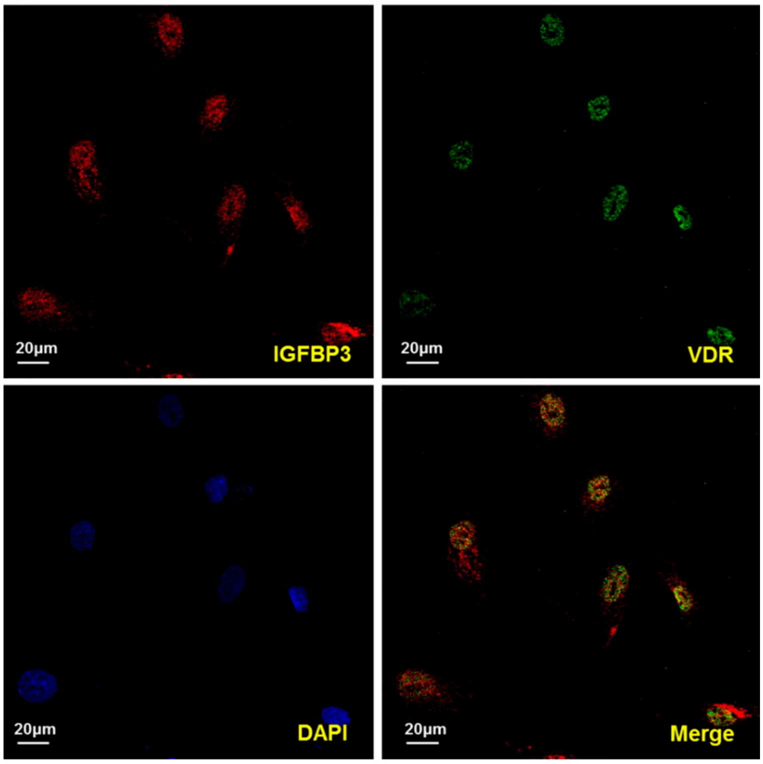


**Figure S3.** *Cont.*

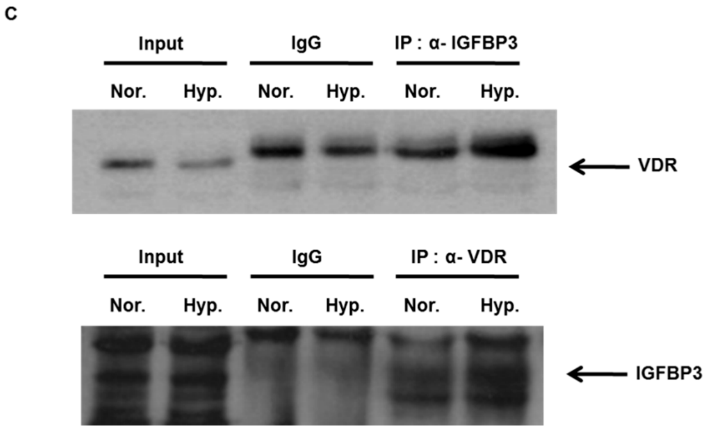


**Figure S3.** Interaction of IGFBP3 and VDR in normoxia and hypoxia. (**A**) ASCs were stained with IGFBP3 antibody (Red), VDR antibody (Green) and DAPI (Blue) for nuclear in normoxia; (**B**) ASCs were stained with IGFBP3 antibody (Red), VDR antibody (Green) and DAPI (Blue) for nuclear under hypoxia; (**C**) IGFBP3 and VDR do not bind in immunohistochemistry.

## 4. Effect of Mitogenic and Hair Regeneration in IGFBP3 Overexpression

IGFBP3 expression level were increased by lenti virus contain *IGFBP3* gene. To investigate mitogenic effect, ASCs were transducted by lenti virus. Cell proliferation decreased in IGFBP3 overexpression. Also, we performed in vivo study for hair regeneration. IGFBP3 did not accelerated hair growth in IGFBP3 overexpression group.

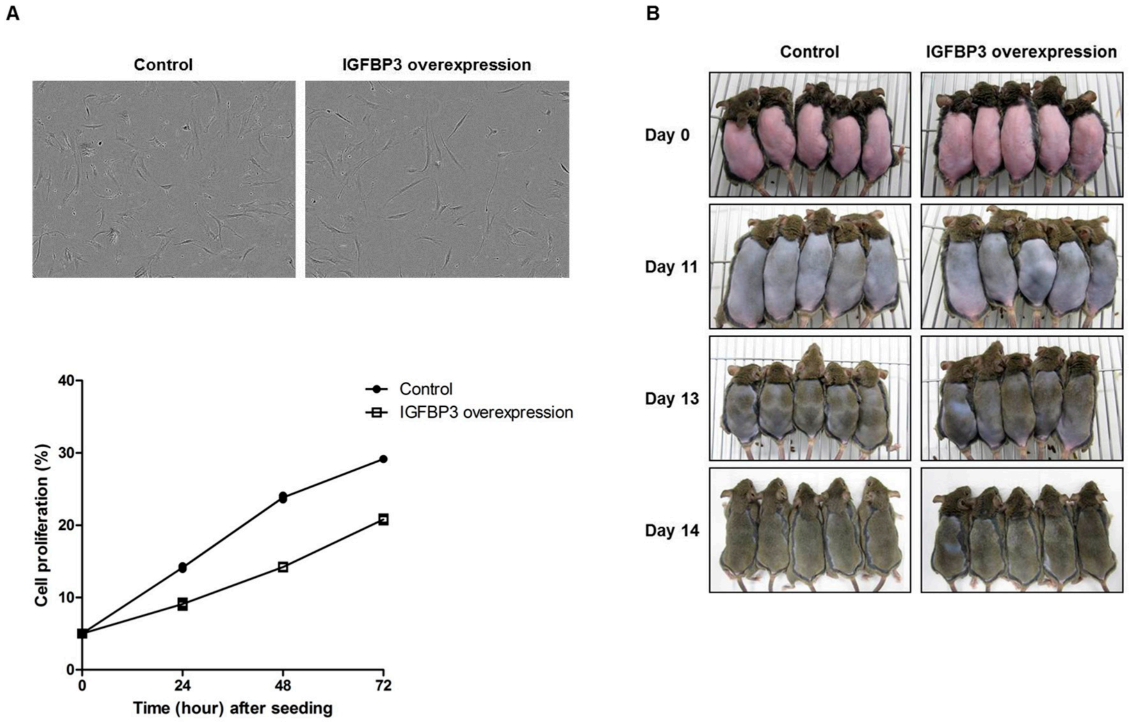


**Figure S4.** Effect of proliferation and hair regeneration in IGFBP3 overexpression. (**A**) ASCs were transducted by lenti virus and obtained cell growth for 72 h (40×); (**B**) IGFBP3 overexpressed ASCs were injected in C3H/HeN mice (7 week old) back skin.
